# Usage of an online tool to help policymakers better engage with research: Web CIPHER

**DOI:** 10.1186/s13012-015-0241-1

**Published:** 2015-04-23

**Authors:** Steve R Makkar, Frances Gilham, Anna Williamson, Kellie Bisset

**Affiliations:** Sax Institute, Level 13, Building 10, 235 Jones Street, Ultimo, NSW 2007 Australia

**Keywords:** Health policy, Research, Websites, Knowledge Platforms, Portals, Innovations, Policymakers

## Abstract

**Background:**

There is a need to develop innovations that help policymakers better engage with research in order to increase its use in policymaking. As part of the Centre for Informing Policy in Health with Evidence from Research (CIPHER), we established Web CIPHER, an online tool with dynamic interactive elements such as hot topics, research summaries, blogs from trusted figures in health policy and research, a community bulletin board, multimedia section and research portal. The aim of this study was to examine policymakers’ use of the website, and determine which sections were key drivers of use.

**Methods:**

Google Analytics (GA) was used to gather usage data during a 16-month period. Analysis was restricted to Web CIPHER members from policy agencies. We examined descriptive statistics including mean viewing times, number of page visits and bounce rates for each section and performed analyses of variance to compare usage between sections. Repeated measures analyses were undertaken to examine whether a weekly reminder email improved usage of Web CIPHER, particularly for research-related content.

**Results:**

During the measurement period, 223 policymakers from more than 32 organisations joined Web CIPHER. Users viewed eight posts on average per visit and stayed on the site for approximately 4 min. The bounce rate was less than 6%. The Blogs and Community sections received more unique views than all other sections. Blogs relating to improving policymakers’ skills in applying research to policy were particularly popular. The email reminder had a positive effect on improving usage, particularly for research-related posts.

**Conclusions:**

The data indicated a relatively small number of users. However, this sample may not be representative of policymakers since membership to the site and usage was completely voluntarily. Nonetheless, those who used the site appeared to engage well with it. The findings suggest that providing blog-type content written by trusted experts in health policy and research as well as regular email reminders may provide an effective means of disseminating the latest research to policymakers through an online web portal.

## Background

The process of using the best available research evidence to inform decision making in health is known as Evidence-Informed Health Policymaking (EIHP) [1,2]. EIHP is purported to lead to better health policies, more effective implementation and more efficient use of resources, with the ultimate goal of improving health outcomes for the wider community [3-5]. Research has informed policies that have improved health in areas such as smoking, alcohol use, immunisation, falls prevention, cardiovascular health, neural development and mental health [6-12]. Evidence indicates, however, that many opportunities to use research to inform health decision making are currently missed [4,6,13-23]. This is, in part, due to various barriers to EIHP such as poor dissemination of research to policymakers [24,25]; deficits in policymakers’ skills in accessing, appraising and applying research to policy [25-28]; a lack of relevant, timely, actionable and locally applicable research [25,28,29]; negative perceptions of the value, quality and usefulness of research held by policymakers [18,30-32]; and an absence of organisational tools and processes to support research access, generation, dissemination and use in policymaking [25,31,33].

There is a need for innovative tools to help policymakers overcome these barriers to accessing research and improve their engagement with research in policymaking. Numerous organisations are now utilising IT-enabled (often web based) tools such as *Knowledge Platforms* in order to improve the ways in which information and knowledge is accessed, stored, retrieved and distributed across organisations [34-38]. It is likely that web-based Knowledge Platforms could be used to overcome some barriers to EIHP, notably difficulties in accessing and disseminating up-to-date, credible and relevant research information to policymakers [31,36].

The Centre for Informing Policy in Health with Evidence from Research (CIPHER) is a Centre for Research Excellence funded by the Australian National Health and Medical Research Council. One of its chief aims is to develop and test new strategies to increase research use in policy. In keeping with this, Web CIPHER (http://www.cipher.org.au) was established by the centre as an online resource to help health decision makers better use, access and engage with research in their work, based on evidence that suggests internet channels can play a role in this [39,40]. Web CIPHER, like other Knowledge Platforms, uses web/email technology to provide users with an efficient means of accessing and delivering comprehensive and up-to-date information regarding health research and policy. Web CIPHER also provides a forum where policymakers and researchers can interact to share knowledge and build on each other’s capacities [35].

**Figure 1:**
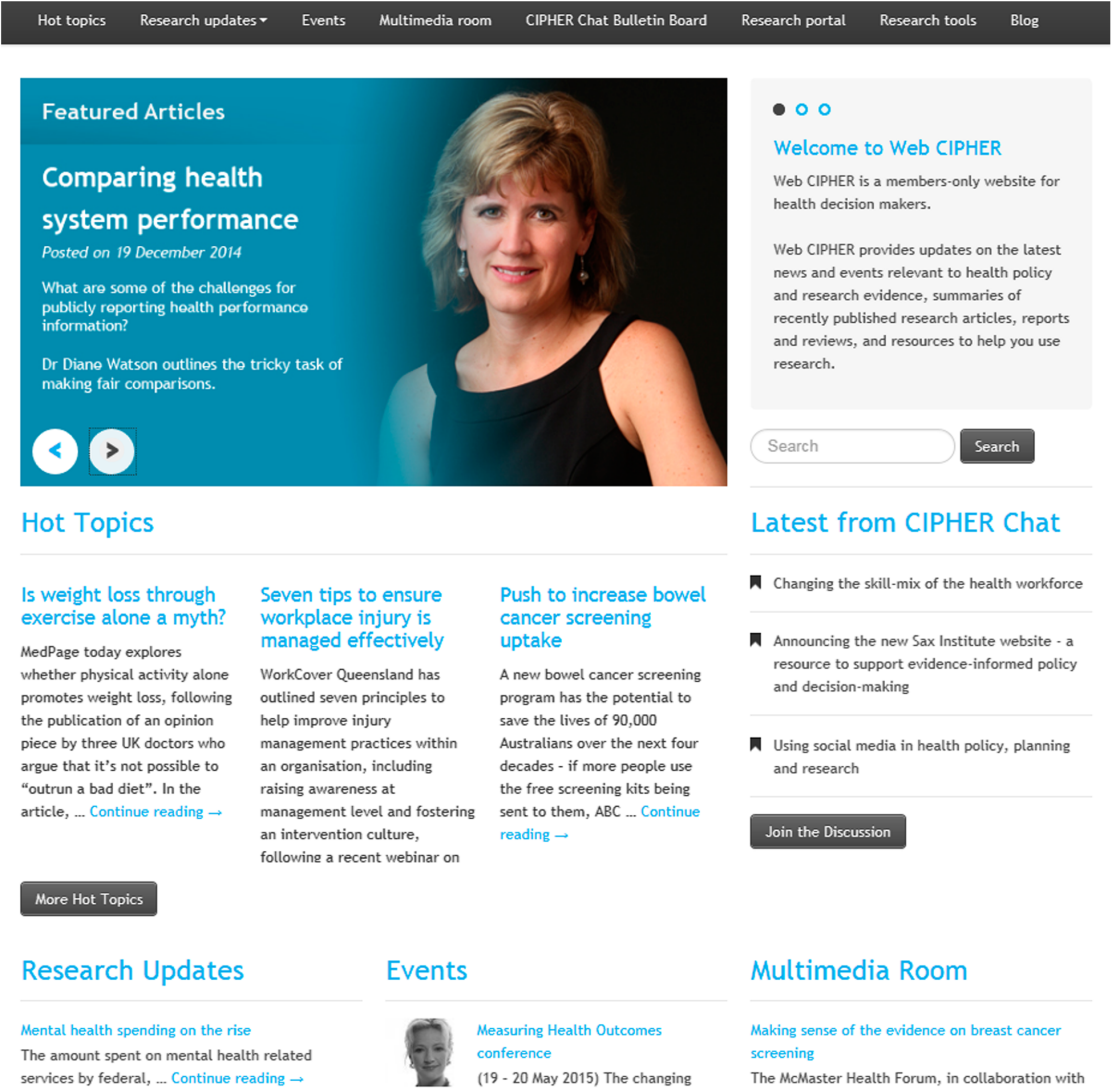
Web CIPHER front page.

Web CIPHER is a password-protected site that contains five main sections with regularly updated content: Hot topics, Research updates, Events, Multimedia and Blog (See Figure 1). It also provides static information in a Research portal section, such as links to systematic review databases and reliable sources of health data and statistics. Information about the general features of Web CIPHER and each specific section is described below. The main features of each section are summarised in Table 1.

**Table 1:** **Details of key sections in Web CIPHER**

**Section**	**Update frequency**	**Content**	**Selection criteria for content**
Hot topics	Daily	Short blurbs linking to news stories relevant to health policy	Media monitoring with keywords of interest set up via Google Alerts. Keywords include the following: “health quality and safety”, “aboriginal health”, “evidence in policy”, “research evidence”, “health and medical research”, “e-health”, “linked health data”, “ageing”, “public health”.
			Daily scan of these alerts for relevant stories of interest to the network, with an emphasis on intervention research, program reviews, locally-relevant news, or examples of evidence-based policy.
Research updates	Fortnightly	Summaries of systematic reviews, research papers and reports, with links to full articles	Fortnightly scan of relevant journals and sources for systematic reviews, research articles and reports.
			Journals include the “Medical Journal of Australia”, “Milbank Quarterly”, “Australian and New Zealand Journal of Public Health”, “Journal of Health Services Research”.
			Other sources include email alerts from the Australian Healthcare and Hospitals Association, the Australian Commission on Safety and Quality in Health Care, Australian Policy Online, the NSW Agency for Clinical Innovation, the National Health and Medical Research Council, the Australian Research Council, the Primary Health Care Research Information Service, the Bureau of Health Information and the Australian Institute of Health and Welfare and the Cochrane Collaboration.
			Sources are reviewed for research, reviews and reports of interest, with an emphasis on public health intervention research, program evaluation, locally-relevant research and research translation.
Events	Monthly	Summaries of upcoming events of interest to health policymakers	Monthly scan of organisations of interest for relevant events, including the Australian Healthcare and Hospitals Association, the Australian Commission on Safety and Quality in Health Care, Australian Policy Online, the NSW Agency for Clinical Innovation, the National Health and Medical Research Council, the Australian Research Council, the Primary Health Care Research Information Service, the Bureau of Health Information, the Australian Institute of Health and Welfare and the Cochrane Collaboration.
			Also reviewed are organisations represented by members, in particular those listed on this page: https://cipher.org.au/the-web-cipher-community/.
Multimedia	Monthly	Videos, audio recordings and presentations of interest to health policymakers	Monthly scan of email alerts from organisations: the Australian Healthcare and Hospitals Association, the Australian Commission on Safety and Quality in Health Care, Australian Policy Online, the NSW Agency for Clinical Innovation, the National Health and Medical Research Council, the Australian Research Council, the Primary Health Care Research Information Service, the Bureau of Health Information, the Australian Institute of Health and Welfare and the Cochrane Collaboration.
			Also scanned are organisations represented by members, in particular those listed on this page: https://cipher.org.au/the-web-cipher-community/.
Blogs	Monthly-quarterly	500-word conversational-style articles written by health leaders, focusing on their area of expertise, with lessons for policymakers	Quarterly meetings with management team to agree on potential topics and contributors. Pitch sent to contributor, and article written by contributor in consultation with senior staff member at the Sax Institute to ensure tone and style matches Web CIPHER requirements.
Community	As required	Bulletin board where users can post information for other community members, such as new research, jobs or opportunities	User-driven.
Research portal	As required	Links to sources of high-quality research and data, such as the Cochrane Library, the Campbell Collaboration, health-evidence.ca, Eppi-CENTRE, NIHR Centre for Reviews and Dissemination, the Australian Institute of Health and Welfare and the Australian Bureau of Statistics.	Updated as required only. Sources are those identified as high quality by CIPHER investigators and Web CIPHER users.

### Hot topics

This section contains short blurbs, posted on the site daily, which link to news stories relevant to health policy and the application of research in health policy. Hot topics aim to provide users with digestible and accessible summaries of the latest developments in health policy and research [21,25,27,30,32,41-43]. The content aims to be engaging, appealing and highly relevant to the Australian policy context [18,26,32,44]. For example, numerous articles focus on health issues related to key subgroups (such as the elderly, children or Indigenous Australians) of interest to the policy agencies in the Web CIPHER community. Many articles also highlight the importance and value of evidence-informed decision making.

### Research updates

This section contains clear, concise, jargon-free summaries, posted fortnightly, of new systematic reviews, research papers and reports, with links to full articles. The summaries are presented in the style of a newspaper report to help users easily comprehend the key findings of each research paper and hopefully encourage them to access the full article. The research articles in this section include primary research studies and systematic reviews covering topics from EIHP to evidence-informed interventions and patterns of diseases and other health conditions of relevance to the agencies in the Web CIPHER community. Primary research articles are included in addition to reviews to provide users with up-to-date, locally and topically relevant information for which systematic reviews have yet to be produced. Also included here are articles on things like evaluations of specific policies, which do not necessarily lend themselves to systematic review.

### Events

This section summarises upcoming events of interest to health policymakers, such as conferences, workshops, seminars or symposia. Policymakers and leaders can use this section to find out about programs to improve their research skills as well as conferences and seminars to learn more about the value of evidence-informed decision making [20,45-47]. This section also alerts policymakers to conferences and symposia where they can gain direct access to the latest research findings in their particular field and establish connections (or perhaps, ongoing partnerships) with researchers [14,48,49].

### Blogs

Blogs contain 500-word conversational-style articles written by health leaders, focusing on their area of expertise. The blogs provide users with a conversational, engaging, reliable and concise presentation of research findings and the application of research to health policy [21,25,27,30,32,41-43]. Many of the blog posts are geared towards improving policymakers’ perceptions of the value of EIHP and providing policymakers with first-hand guidance and advice on strategies to improve research use in decision making [20,27,45].

### Multimedia

This section contains videos, audio recordings and presentations of interest to health policymakers. In many of the videos and audio files, internationally recognised and trusted health researchers present research findings, discuss innovations and strategies to integrate research into policy, as well as the value of EIHP. The use of a variety of media formats facilitates dissemination of research findings and presents research-policy information in an accessible and engaging format to users [28,44].

### Community

This section contains a Bulletin Board where users can post information for other community members, such as new research, research-policy events like conferences or symposia, new job opportunities or other relevant updates. The Bulletin Board can thus be used by policymakers and researchers to access research, disseminate relevant research studies to other policymakers and discuss its applicability to current policy decisions [14,25,49].

### Research portal

The research portal is a static section that provides users with web links to sources of high-quality research and data, such as the Cochrane Library. There are links to websites that offer one-stop shopping for systematic reviews, evidence briefs, high-quality research journals and databases and reliable sources of data and statistics (e.g. Australian Institute of Health and Welfare). The Research portal is especially helpful to policymakers if their organisations do not provide tools and systems to assist them in searching for and accessing research findings [45,48].

### Additional features

Users who become members of Web CIPHER are sent a weekly summary email that provides links to the latest Hot topics, Research updates, Blogs and Multimedia added to the site during the week. This email provides an effective means of disseminating relevant research to policymakers and improving their access to such research [45,48].

Finally, Web CIPHER also includes a search bar and tags (such as “Obesity”, “Nutrition”, “Aboriginal and Torres Strait Islander Health”) that allow users to easily search for and access research articles and summaries, hot topics and other posts on topics of interest.

Web CIPHER aims to help policymakers access and engage with research more effectively, but these benefits are dependent on users accessing and using the website. Consequently, we conducted a pilot study analysing preliminary usage data to identify which sections were most appealing to users and what features of Web CIPHER were particularly effective at driving usage. We intend to use this information to guide future strategies to improve policymakers’ use and engagement with the website.

## Method

### Sample

A large number of state and federal-level health agencies were formally invited by the CIPHER chief investigators to attend the official launch of CIPHER. These agencies were invited on the basis that they routinely developed health policies, programs and practises and were based at the state or federal level. Following the launch, executives from these agencies were formally invited by email to join the CIPHER network. Employees from those agencies interested in becoming CIPHER members were then invited by email to join Web CIPHER.

As part of applying to join the site, applicants were required to report their place of work. Users were accepted as members if they reported working for one of the CIPHER network agencies or any other Australian organisation engaged in health policy or program work. For this pilot study, the target sample thus consisted of health decision makers, practitioners or researchers (primarily heads of research institutes or schools) from organisations within the CIPHER network.

### Data analysis

Following 16 months of use, we used Google Analytics (GA) to obtain site usage data for the period of 1 January 2012 to 30 April 2014 inclusive (see Table 2). Using the place of work, we created a custom segment made up of Web CIPHER members from policy agencies only (excluding members from universities, the Sax Institute and the website developers). We exported data for each post and manually coded each post according to its section: Research updates, Events, Hot topics, Multimedia, Blogs and Community. The Community section included the Bulletin Board and other posts describing the policy agencies participating in the CIPHER Community.

**Table 2:** **Mean usage and standard deviations for Web CIPHER sections and other select pages**

**Page type**	**Total number of posts**	**Total number of unique post views**	**Minimum views for a single post**	**Maximum views for a single post**	**Unique page views**		**Average viewing time (s)**			
					***M***	**SD**	***M***	**SD**	***M***	**SD**
Research	132	1275	1	97	4.69	10.89	32.89	60.19	0.00	0.06
Events	34	273	1	52	4.27	8.86	31.58	52.26	0.00	0.00
Hot topics	347	1996	1	199	3.25	9.40	43.03	82.55	0.00	0.04
Multimedia	36	260	1	40	3.33	6.35	23.16	42.28	0.00	0.00
Blog	20	759	1	110	12.65	17.87	43.46	57.18	0.00	0.00
Community pages	42	902	1	138	11.87	20.33	27.37	50.29	0.00	0.00
Research portal^a^	1	124	124	124	124.00		46.47		0.00	
Skills posts					7.57	16.67	41.08	57.40	3.87	13.84
Non-Skills posts					7.19	61.42	37.63	84.02	2.49	45.55
Research Value posts					6.06	13.14	67.27	196.72	0.00	0.00
Non-Value posts					7.29	62.32	35.58	67.06	0.00	0.06
Posts accessed directly from email link					14.88	6.32				
Posts accessed indirectly from an email link					10.95	11.02				
Accessed from an alternative route					8.81	37.31				

^a^Minimum number of page views, means and standard deviations for the Research portal page cannot be computed because it consists of only a single page.

We conducted a series of statistical analyses using the GA data. First we calculated basic usage statistics for the site overall and individual sections (e.g. Research updates, Hot topics, Blogs). We then conducted multivariate analysis of variance (MANOVA) with follow-up univariate tests and Scheffe comparisons to compare usage between key sections. Usage was defined by three indicators: (i) number of unique visits to each page, (ii) average time on page in seconds, and (iii) page bounce rate (i.e. percentage of visits where users left the site immediately after viewing that particular page).

Statistical analyses were then conducted to examine usage for pages with similar topics across all sections. We examined usage for the following: (i) posts relating to improving research skills and/or the use of research in policy (*Skills* pages) and (ii) posts focused on promoting the value of incorporating research into policy (*Value* pages). These pages were identified and coded by the first author (SM). MANOVA was conducted to compare usage between Skills/Value pages and other post types (Hot topics, Blogs, Research updates, Events or Multimedia).

Finally, we conducted repeated measures analyses to examine whether the weekly Web CIPHER email improved the ability of users to access the posts on the website. Specifically, we compared the level of usage between the same page accessed (i) *directly* via an email link, (ii) *indirectly* (the page was visited from another page accessed by an email link) or (iii) an *alternative* route (e.g. the Sax Institute website, a search engine or typing in the address directly). In total, there were 303 posts advertised in the weekly Web CIPHER email throughout the study period. To conduct this analysis, each post was examined separately in GA and manually coded to evaluate the number of times individuals accessed that post through each of the above-mentioned access routes. These analyses are described in detail below. Means and standard deviations for the usage data are displayed in Table 2.

## Results

### Sample

A total of 223 policymakers from 27 organisations joined Web CIPHER during the 16-month study period. Six of the organisations accounted for more than 60% of the total target sample. Users held a diverse range of positions within their organisations, although the most commonly reported were directors or deputy/assistant directors (13%), managers (24%), project officers/coordinators (17%), policy officers, advisors, or analysts (12%) and research officers/fellows (7.6%). Other demographic information such as age and sex was not collected from participants.

### General usage

General usage data revealed that during the 16-month time period analysed, users viewed 8 posts on an average visit, staying on the site for approximately 4 min. The bounce rate was just over 6%.

### Usage of Web CIPHER sections

#### Main sections

##### Descriptive statistics

Data revealed that for the 272 Research updates posts on Web CIPHER, each received an average of five unique post views, an average viewing time of 33 s and a bounce rate of less than 1%. Events posts had about four unique post views, an average viewing time of about 31 s and a bounce rate of less than 0.1%. Hot topics and Multimedia posts each had close to three unique post views and very low bounce rates (each less than 0.1%). Each Hot topics post was viewed for about 43 s, and Multimedia posts were viewed for about 23 s. Posts within the Blog section of the website were the most popular, receiving on average almost 13 unique views per post, a bounce rate of less than 0.1% and an average viewing time of 43 s per post. Community posts were viewed an average of 12 times and for 27 s each. The bounce rate again was less than 0.1%. It should be noted that there were only five posts from non-Sax Institute members on the Bulletin Board, indicating that the Community section was virtually unused by members. The Research portal page received 124 total unique post views, with an average viewing time of 46 s and a 0% bounce rate.

##### Comparisons

The Research portal section was not included because it was only a single static page. The analyses revealed a significant multivariate main effect of post type. Scheffe follow-up comparisons showed that posts in the Blog section received a higher average number of unique views than all other types of posts (*p* < .05 for each section comparison) except Community posts (*p* > .05). There were no significant differences between Research updates and Multimedia, Events or Hot topics posts in terms of post views, viewing time or bounce rate; between Events posts and Hot topics or Multimedia; or between Hot topics and Multimedia (all *p*s > .05).

Despite the lack of posts on the Bulletin Board, the Community section had a significantly greater number of unique views relative to posts in the Hot topics, Research updates, Events, and Multimedia sections (*p* < .05 for each comparison). Therefore, Blogs and Community posts may provide the most effective means of disseminating research to users.

### Usage of pages on a specific topic

#### Pages relating to improving research skills

Fifty three Skills pages were identified. Descriptive statistics revealed that each Skills post had, on average, 7.57 unique post views, with a bounce rate of less than 1% and an average post viewing time of 41.08 s. Multivariate analyses did not reveal any significant differences between Skills posts and other post types.

Since Blogs was the most popular section, we conducted an exploratory two-way MANOVA to see whether Blogs focused on Skills were more popular than either Skills or Blogs posts on their own. The two-way MANOVA revealed a non-significant main effect for Skills posts, *F*(1, 1264) = 1.68, *p* = .14, η_p_^2^ = .01, and a significant main effect for Blogs posts, *F*(5, 1264) = 5.40, *p* < .01, η_p_^2^ = .02. Interestingly, however, the Skills by Blog interaction effect approached significance, *F*(5, 1264) = 1.98, *p* = .08, η_p_^2^ = .01. Examining univariate tests revealed that this interaction effect was marginally significant for average viewing time per post, *F*(5, 1268) = 3.09, *p* < .08, η_p_^2^ = .002. This indicates that although Blogs received longer average viewing times relative to other posts, this effect was greater for Skills posts than non-Skills posts. In other words, viewing times were greater for posts that combined Skills with Blogs (*M* = 79.12 s, SD = 23.97), compared to Skills (*M* = 29.93 s, SD = 12.97) or Blog posts alone (*M* = 34.53 s, SD = 11.99). These findings suggest that Blogs may provide an effective means of conveying advice and building policymakers’ research skills.

#### Pages related to the value of research in health policy

Eighty-eight Value pages were identified. Descriptive statistics revealed that each Value-type post had, on average, six unique post views, with a bounce rate of less than 1% (*M* < .001, SD = .002) and an average post viewing time of 41.08 s (SD = 67.27). Multivariate analyses comparing Value pages to other posts revealed a multivariate main effect, *F*(5, 1266) = 3.102, *p* < .01, η_p_^2^ = .01. Univariate tests revealed viewing time was significantly greater for Value posts relative to other posts. The findings suggest that policy maker members of Web CIPHER are quite engaged with posts that support the use of research in policymaking.

### Impact of the weekly Web CIPHER email on usage

Repeated measures analysis of all Web CIPHER posts that were delivered via the weekly newsletter email, revealed a significant main effect of access route, *F*(2, 301) = 5.34, *p* = .005, η_p_^2^ = .03. Scheffé comparisons showed that posts received significantly more unique views via a *direct email link* or *indirect email link* relative to an *alternative* route (all *p*s < .01; these access routes are described in the ‘Method’ section). However, the number of unique views did not differ between direct and indirect email links (*p* = .16). A similar result emerged when we just focused on research-related posts (i.e. posts from within the Research updates and Hot topics section), although this time, posts received significantly more unique views via a direct link, relative to an indirect link (*p* < .01).

An exploratory analysis was undertaken to determine if the effect was greater for *Research posts* (Research updates and Hot topics posts) relative to *Other posts* (Blogs, Community, Multimedia, Events). A 2 (type of post: Research versus Other) × 3 (access route: direct email link, indirect, alternative) MANOVA revealed a significant main effect of post type.

**Figure 2:**
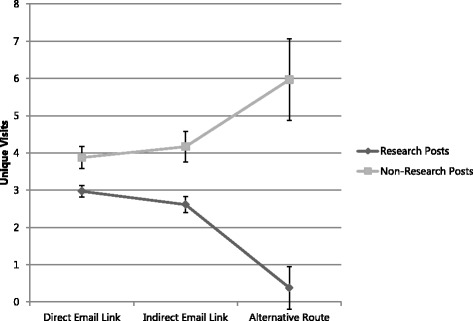
Unique visits for Research and Non-Research Posts accessed directly via an email link, indirectly from another post accessed by an email link or through an alternative route.

Of most interest was the significant route by post type interaction, *F*(2, 300) = 8.22, *p* < .001, η_p_^2^ = .05, which indicated that email delivery was more helpful in increasing unique views of Research posts, relative to Other posts (see Figure 2). Analysing this interaction in greater detail, simple effect analyses revealed that the access route had no impact on views for Other posts, *F*(2, 300) = 2.08, *p* = .18, η_p_^2^ = .01. In contrast, access route had a significant impact on unique views for Research posts, *F*(2, 300) = 11.75, *p* < .001, η_p_^2^ = .07. What this means is that the weekly Web CIPHER email was effective in increasing views of Research posts (i.e. Research updates and Hot topics posts), but not Other posts. This suggests that a weekly summary email increases the likelihood that policymakers access and view the latest health policy research and updates.

## Discussion

Our findings suggest that Web CIPHER has the potential to increase the extent to which policymakers engage with research. Usage statistics revealed that for the 223 site members, average viewing time was greater than 35 s, the bounce rate was well below 5% in all sections and users typically viewed more than one page per visit. These statistics are positive given that the average bounce rate for most websites falls within 40%–60% and that website users, on average, leave web pages within 10–20 s [50].

Online Knowledge Platforms are increasingly being used by organisations to improve performance and best practise by overcoming barriers relating to information access, storage, retrieval and exchange [35-37]. Evidence indicates that effective Knowledge Platforms (i) are easy to use and (ii) provide comprehensive, up-to-date, relevant and easily accessible information [34,38]. Web CIPHER aims to apply these features in order to improve the extent to which health policymakers engage with research. The presentation of relevant research in multiple formats (e.g. summaries, blogs, multimedia, full-text articles) through an easy-to-use website and a weekly email was designed to ensure research findings were easily accessible, appropriately disseminated, digestible, engaging, timely and relevant to the local context. Because membership to Web CIPHER is free, there were no financial and only a few organisational barriers to accessing and using the site over the study period.

Of greatest interest was the result demonstrating that Blogs and Community posts were the most popular sections on the site. This indicates users were keen to read the points of view of experts in health policy and research. Blog posts were helpful in increasing viewing time for research skill-related posts (relative to non-skill-related posts). This indicates that blog posts may provide a medium for conveying advice to policymakers about ways to integrate research into policy, which may to some extent address a key barrier to EIHP: policymakers not having the skills to access, appraise and apply research to policy [25,31,32,51].

There are numerous possible explanations as to why the Blogs were particularly popular. They were written by trusted figures in health policy and research. This increased sense of trust and credibility may have motivated users to not only read the Blogs, but to access the other key sections of the website. Research suggests Knowledge Platforms are more likely to be utilised by staff if perceived as credible and championed by trusted and senior individuals [34,35]. Having blog posts written by trusted figures may also have contributed to a sense of community, trust, and social presence on the site, all of which are key predictors of website use [52-56]. Further, the fact that Blogs were written by familiar and trusted figures could potentially overcome negative attitudes reportedly held by some policymakers towards the quality and credibility of research and researchers [24,27].

The Blogs in Web CIPHER were also written in a way that addresses some of the research needs of policymakers [31]. The authors strove to write Blogs in a non-intimidating and conversational tone, with minimal use of jargon. They were presented in a clear, concise and easy-to-use format, akin to the style of newspaper reports or editorials. Importantly, they often summarised relevant research findings and provided clear and concrete recommendations to policymakers. Numerous studies indicate that these features are key facilitators of evidence-informed health policymaking [18,26-28,57]. Furthermore, research in website usage has shown that the perceived ease of use of a website and the usefulness of the information it provides are significant predictors of its use [58-60]. In sum, Web CIPHER Blogs were written in a way that addressed many of the aforementioned barriers to research use such as poor formatting and presentation of research findings, excessive use of jargon, limitations in policymakers’ research skills and a lack of clarity, timeliness and policy relevance of research [25,31,51].

The fact that Community Bulletin Board posts also received a relatively high number of unique views compared to other sections indicates that members are keen to read about the points of view of their colleagues and contemporaries. Unfortunately, though, few members posted on the Bulletin Board. Nonetheless, our findings indicate that blogs and bulletin board posts may provide an effective means of conveying or disseminating the latest research, providing advice regarding evidence-informed health policy, building policymakers’ research skills and improving their perceptions towards research use in policy.

In light of these findings, we intend to test strategies to improve Web CIPHER’s capacity to improve policymakers’ engagement with and use of research in policy. Specifically, we aim to capitalise on the most popular feature of the site by increasing the number of blog posts. Further, we will test the impact of strategies such as having leaders from Web CIPHER’s member agencies contribute to these blogs on use of the site amongst other agency staff. Since blogs may provide an effective conduit for engaging users with research, we also intend to link relevant research posts directly to these blogs to determine the impact of this on research post views.

Another key finding was that posts, particularly research-related, were most likely to be accessed from a link provided in the weekly summary email. This reinforces the importance of improving access as a way to facilitate research engagement [21,25,27,30,32,41-43,48,61]. An effective means of building on this strategy may be to invite agency leaders to occasionally guest author the weekly emails sent to Web CIPHER members alerting them to the latest Web CIPHER research and information updates. It is hoped that these leaders and nominated staff will then be seen as champions of research use by the Web CIPHER community. Indeed, studies indicate that use of Knowledge Platforms is related to the extent to which they are championed by organisations’ senior management [34,38]. Such championing is likely to improve users’ perceptions of trust and informational credibility, relevance, social presence and sense of community [52-56] and may ultimately impact on policymakers’ attitudes towards EIHP.

In order to examine these proposed mechanisms more closely, it will be essential in future researches to document policymakers’ views on whether and how these strategies impacted on their use of Web CIPHER. Furthermore, it will be valuable to investigate whether policymakers’ usage of Web CIPHER has to some extent changed the way in which they perceive and engage with research in policy.

### Strengths and limitations

The current paper has a number of strengths. In particular, it describes the innovative use of Google Analytics to observe the usage of an online knowledge exchange portal to help policymakers engage with research in policymaking and empirically examine which aspects of the portal were particularly appealing to this audience. We incorporated a number of usage metrics (unique views, average time on page, bounce rate), analysed and compared usage of key relevant sections of the website, identified the most appealing content on the site and evaluated the positive impacts of delivering research content directly to users via email. These analyses have shed light on promising strategies to improve user engagement with Web CIPHER and policymakers’ perceptions towards EIHP. We hope this will be helpful to others as we are not aware of any other research that describes effective ways for organisations to set up similar knowledge exchange portals. We believe, therefore, that our paper makes a significant contribution to understanding the potential benefits of such online portals and potential key strategies that can be implemented to enhance their potential for improving policymaker engagement with research.

Limitations primarily concern the sample on which the analyses were based. Firstly, the number of registered users was relatively small (*n* = 223). These small numbers were likely impacted by the site’s requirement for users to apply, obtain approval to join the site and to be logged in with a username and password to view content. This was a requirement for analysis, although it is certain to have posed a barrier to site use. Further, as Web CIPHER was still in its pilot phase, access had only been offered to a limited number of Australian policy agencies.

Selection bias may also be an issue for our study sample. Specifically, because membership (and usage) was completely voluntary, our sample was essentially self selected and may have primarily consisted of policymakers with pre-existing interests in research or knowledge exchange. Therefore, our conclusions regarding usage and suggested improvements are necessarily preliminary. Randomised controlled trials would allow us to make more definitive conclusions about what components of web portals are particularly effective at driving usage and improving how policymakers engage with research.

It is possible that the findings observed in the present study are specific to the time frame in which observations were made (i.e. the 16-month period from when the site was first launched in January 2012 until April 2014). Google Analytics data indicates there has been gradual growth in the number of Web CIPHER users after this period. It is possible that the distribution of users will change leading to variations in site usage. It will be important to utilise a longer observation period in future research in order to verify the findings observed in the present study.

There are also limitations to the use of Google Analytics metrics to assess usage, in particular *unique* page views. A unique page view occurs when a user visits a page in one viewing session. If the user visits the page more than once in a session, it is still only counted as a single unique page view. However, unique page views may be the result of multiple users viewing a page, or a single user visiting the same page across multiple sessions. Therefore, unique views alone cannot accurately indicate the popularity of particular pages or the extent to which they are used by multiple users. It is therefore essential that other metrics are incorporated, which is why we also reported average page viewing time and bounce rate (i.e. the percentage of visitors that view only a single page before exiting the site). These two indices can provide additional indications of the extent to which users are interested with the site [50]. Nonetheless, it is important to note that such online metrics only provide, at best, an estimate of the extent of usage of a particular website.

## Conclusion

Like other Knowledge Platforms, Web CIPHER has the potential to improve information access, dissemination and exchange in order to improve policymakers’ engagement with research in policymaking. The current study makes a useful contribution to the literature by providing insights into some of the most effective features of such sites in a health policy environment. Our findings revealed that users were generally engaged with Web CIPHER content, and were particularly drawn to the Blogs and Community sections of the site. The Blogs may have been particularly appealing to users because they addressed topical issues presented by trusted figures in health policy and research and were written in a conversational and engaging tone. A weekly reminder email increased the likelihood that users accessed site content, particularly those posts relating to research. The findings suggest that online knowledge exchange portals may improve policymakers’ engagement with research by incorporating topically relevant blogs written by trusted figures in health policy and research and utilising a weekly reminder email to facilitate access to this content.
